# Diagnostic Value of Pelvic MRI for Assessment of the Depth of Myometrial Invasion and Cervical Involvement in Endometrial Cancer: Comparison of New Versus Old FIGO Staging

**DOI:** 10.5812/iranjradiol.5276

**Published:** 2012-11-20

**Authors:** Fatemeh Zamani, Shirin Goodarzi, Faride Hallaji, Azadeh Zamiri, Tourisa Deilami, Mahrooz Malek, Mitra Modarress Gilani

**Affiliations:** 1 Department of Radiology, Tehran University of Medical Sciences, Tehran, Iran; 2 Department of Gynecology Oncology, Vali-e-Asr Hospital, Tehran University of Medical Sciences, Tehran, Iran; 3 Department of Radiology, Medical Imaging Center, Advanced Diagnostic and Interventional Radiology Research Center (ADIR), Imam Khomeini Hospital, Tehran University of Medical Sciences, Tehran, Iran

**Keywords:** Endometrial Neoplasms, Uterus, Magnetic Resonance Imaging, Myometrium, Cancer Staging

## Abstract

**Background:**

Endometrial carcinoma is a highly prevalent gynecologic malignancy. The International Federation of Gynecology and Obstetrics (FIGO) staging system underwent significant revision on 2009. Key changes in the FIGO staging system include simplification of stage I endometrial cancer and removal of cervical mucosal invasion as a separate stage. MRI is a noninvasive diagnostic method for preoperative staging of endometrial cancer.

**Objectives:**

The main purpose of this study was to investigate the diagnostic efficacy of pelvic MRI in determining the depth of myometrial invasion and cervical involvement in endometrial carcinoma. The other aim was to compare the accuracy of pelvic MRI using the old and new FIGO staging systems in endometrial carcinoma.

**Patients and Methods:**

Between November 2010 and January 2012, 54 patients underwent primary surgical staging in our department due to endometrial adenocarcinoma. Pre-operative pelvic MRI was performed and MRI staging was done according to old and new FIGO staging, separately. The sensitivity, specificity, positive and negative predictive values as well as the accuracy of MRI for deep myometrial invasion and cervical infiltration were calculated. MRI accuracy was also compared for old and new FIGO staging. Pathological staging was the standard of reference.

**Results:**

The mean age was 53.31 (SD = 11.52) and the most common histological subtype was the endometrioid type of endometrial adenocarcinoma (90.8%). In the evaluation of deep tumoral invasion of the myometrium (> 50%), sensitivity, specificity, diagnostic accuracy and positive and negative predictive values of MRI were 82.35%, 94.59%, 90.74%, 87.5% and 92.1%, respectively. For cervical stromal involvement, these values were 54.54%, 100%, 90.74%, 100% and 89.58%, respectively. In case of cervical mucosal involvement (in old FIGO staging), the positive predictive value was only 50% and the accuracy decreased to 74.07%. Agreement between MRI and the final histology using the old and new FIGO classification was appropriate with Kappa = 0.62 and 0.72, respectively (P < 0.001).

**Conclusion:**

Using 2009 FIGO classification increases the accuracy of pelvic MR imaging for preoperative staging of patients with early stages of endometrial cancer.

## 1. Background

Endometrial cancer is the most common gynecological cancer. The incidence is growing because of increasing obesity and age (1). One of the most important aspects of successful patient management with endometrial cancer is the accurate staging of the disease at the time of diagnosis and initiating a right treatment plan without causing any more morbidity. Before 1988, staging was performed by examination under anesthesia and D & C. In this method, at least 13-22% of the patients underwent inaccurate staging before treatment planning, which results in a decreased survival (2). In 1988, surgicopathologic International Federation of Gynecology and Obstetrics (FIGO) staging was introduced (Table 1) (3). With reported similar prognosis in some of the FIGO substages (stage Ia and IIa) in 2009, some precise changes occurred in staging (Table 2) (4-6). In the new staging system for stage I, there are two substages Ia (< 50% myometrial invasion) and Ib (≥ 50% myometrial invasion) in comparison with the old staging system in which there are three substages; Ia (confirmed to endometrium), Ib (< 50% myometrial invasion) and Ic (≥ 50% myometrial invasion). There are no more substages for stage II and only cervical stromal involvement (stage IIb, old system) is considered and endocervical glandular invasion (stage IIa, old system) is now diagnosed as stage I endometrial cancer. Differentiation between stage Ia and Ib in the old staging system and also glandular involvement of the cervix had some challenges. It is believed that with the new 2009 FIGO staging, a better accuracy in MRI staging is obtained (7, 8). The information about the role of MRI in endometrial cancer in our country is limited. In this study, we assessed the sensitivity, specificity, positive predictive value (PPV), negative predictive value (NPV) and accuracy of MRI in recognizing the depth of myometrial invasion and cervical involvement. Furthermore, its accuracy in old (1988) and new (2009) FIGO staging classifications is compared.

**Table 1:** 1988 FIGO Staging Classification for Endometrial Cancer

Stage	Characteristics
** Ia G1, 2, 3**	Confined to endometrium
** Ib G1, 2, 3**	< 50% myometrial involvement
** Ic G1,2,3**	≥ 50% myometrial involvement
** IIa**	Endocervical glandular involvement
** IIb**	Cervical stromal invasion
** IIIa**	Invasion to uterine serosa, adnexa and/or positive peritoneal cytology
** IIIb**	Parametrium involvement, vaginal metastasis
** IIIc**	Pelvic or paraaortic lymph nodes involvement
** Iva**	Bladder and/or bowel mucosal metastasis
** IVb**	Distant metastasis including intraabdominal or inguinal nodes

**Table 2:** 2009 FIGO Staging System for Endometrial Cancer (New Staging)

FIGO Stage	Pathologic Finding	MRI Finding
**Stage I^a^^c^**	Tumor confined to the uterus, < 50% myometrial invasion	The endometrial mass is confirmed to the endometrium or invades < 50% of the myometrial wall with disruption of the JZ
**Stage I^b^^c^**	Tumor confined to the uterus, ≥ 50% myometrial invasion	Mass invades ≥50% of the myometrium with a preserved stripe enhancing outer myometrial wall
**Stage II^c^**	Cervical stromal invasion	Disruption of the low-signal intensity inner cervical stroma (T2).
		Disruption of the cervical epithelium enhancement (CE T1)
**Stage III^a^^c^**	Tumor invasion into serosa or adnexa	Disruption of continuity of the outer myometrium or presence of nodules on the peritoneal surface or adnexa
**Stage III^b^^c^**	Vaginal or parametrial involvement	Tumor extension into the upper vagina and/or parametrium
**Stage IIIc1^c^**	Pelvic node involvement	Enlarged pelvic lymph nodes (cut-off value: > 10mm for short-axis diameter)
**Stage IIIc2^c^**	Para-aortic node involvement	Enlarged para-aortic lymph node
**Stage IV^a^^c^**	Tumor invasion into the bladder or bowel mucosa	Tumor extension into the bladder/rectum with disruption of the hypointense signal of the bladder or rectal wall
** Stage IV^b^^c^**	Distant metastases including abdominal or inguinal lymph node involvement	Intra-peritoneal metastases in the peritoneum or omentum
		Distant metastases to the lung, liver or bones. Distant lymph node metastases

^a^Exclusively endometrial glandular involvement should be considered stage I

^b^Positive cytology should be reported separately without changing the stage

^c^Either G1, G2 or G3

Abbreviations: CE; contrast enhanced, FIGO; International Federation of Gynecology and Obstetrics, JZ; junctional zone, T1; T1-weighted imaging, T2; T2-weighted imaging

## 2. Objectives

The aim of this study was to assess the diagnostic accuracy of MR imaging for presumed myometrial and cervical involvement. Meanwhile, we compared the new and old FIGO staging and their agreement with MRI diagnosis.

## 3. Patients and Methods

Our study was performed between November 2010 and January 2012 on 65 women with endometrial cancer. Eleven patients were excluded from the study; six of whom were due to medical contraindications for surgery or advanced disease that needed chemotherapy or radiotherapy before surgery and five patients due to the final carcinosarcoma diagnosis. For MR imaging, the patients fasted for 4-6 hours before the procedure. They voided before MRI to avoid bladder fullness. MR imaging was acquired with 1.5 T system and phased-array pelvic coil. In our protocol, T2-weighted sequences in three planes (axial, axial oblique and sagittal sections of the uterus body) were obtained. The obtained axial oblique images were perpendicular to the endometrial cavity. Native T1-weighted sequences in the sagittal plane were taken too. The later sequence was repeated in axial oblique and sagittal planes with fat suppression before and after IV-contrast administration. Gadolinium was used as the intravenous-contrast in a dose of 0.1 mmol/kg of body weight. Contrast-enhanced MR imaging was not performed in patients with renal impairment (GFR < 30ml/min). MRI findings were reviewed by a radiologist that was aware of endometrial cancer diagnosis, but without knowledge about the surgical and pathological stage of the patients. On MRI images, when the low signal intensity of the junctional zone was preserved, the tumor was limited to the endometrium, so myometrial infiltration was excluded. Concerning the cervix, involvement of endocervical glands and stroma were assessed. Radiological staging was performed according to both new and old FIGO staging systems (Table 2). Surgical specimens were sectioned across the longitudinal plane of the uterus. The depth of myometrial invasion was estimated macroscopically and microscopically without awareness of MR findings and was staged according to FIGO classification. Ultimately, MRI findings were compared with the pathological reports. All patients underwent staging surgery including total hysterectomy and bilateral salpingo-oophorectomy. For suspicious cases, lymphadenectomy was performed. If the cervix was involved, a radical hysterectomy would be done. Statistical analysis was performed by SPSS for Windows 17 (SPSS Inc., Chicago, Illinois, USA). Descriptive statistics, chi-square and Kappa index were used for analysis. The sensitivity, specificity, positive and negative predictive values and accuracy for deep myometrial and cervical infiltration were calculated.

## 4. Results

**Figure 1:**
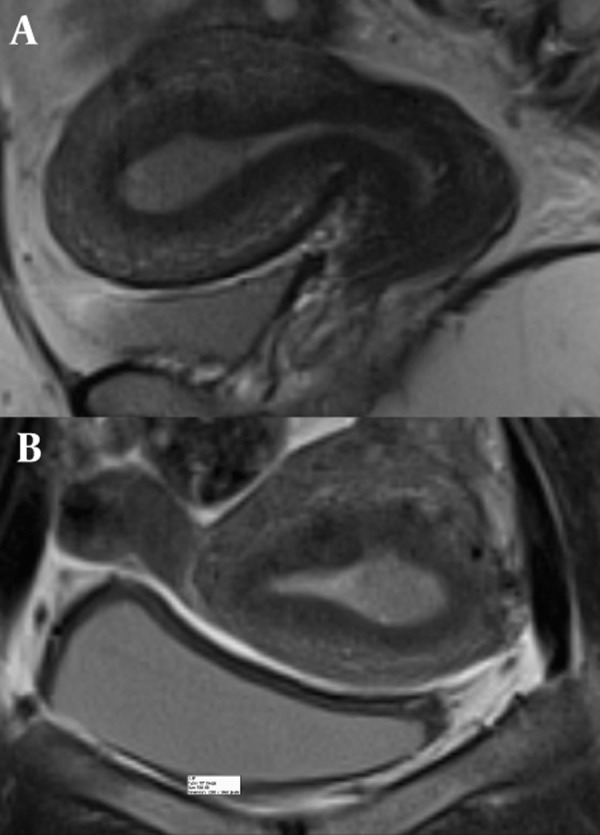
A 40-year-old woman with vaginal bleeding. Sagittal T2-weighted (A) and coronal T2-weighted (B) images show an intermediate signal lesion within the endometrial cavity in A and B. The junctional zone is intact. Histopathology revealed stage Іa endometrial carcinoma.

Among 54 patients included in this study, the mean age was 53.31 years (range 24-73years). The most common chief complaint was post-menopausal bleeding (57.4%) and abnormal bleeding before menopause (33.3%). Pelvic mass, ascites and inguinal lymph node were the first presentation in the other patients. The most common histological subtype was the endometrioid type detected in 90.8% (49) of the patients. Papillary serous, clear cell and adenosquamous types were the other subtypes. The most common grade was well differentiated type (G1) 53.7% (Table 3). In the surgicopathological report, in 51.9% (28/54), the myometrial depth of invasion was less than 50% and in 31.5% (17/54), there was an equal or greater than 50% involvement and nine patients (16.75%) had no involvement of the myometrium. Seven out of 16 patients (43.8%) who did not have any myometrial invasion in the MRI report, had lower than 50% myometrial involvement in the final pathological findings (Figure 1). Three out of 22 patients (13.6%) with less than 50% myometrial involvement in MRI ultimately had more than 50% myometrial involvement. Fourteen out of 16 patients (87.5%) with an MRI report of more than 50% myometrial invasion had the same findings in final pathology reports. In the evaluation of the deep myometrial invasion (more than 50%), the sensitivity, specificity, diagnostic accuracy, positive and negative predictive values and positive and negative likelihood ratios of MRI (calculated with 95% confidence intervals) were 82.35%, 94.59%, 90.74%, 87.5% ,92.1% ,15.22 and 0.1865, respectively. Table 4 shows the correlation of myometrial involvement in MRI and pathology.

**Table 3:** Patients’ Characteristics

Number of women	54
**Mean age in years, range**	53.31(24-73, SD = 11.52)
**BMI, No. (range)**	31.1(17-48, SD = 6.44)
**Chief complaint, No. (%)**	
Post-menopausal bleeding	31(54.7)
Abnormal bleeding before menopause	18(33.3)
Pelvic mass	3(5.6)
Ascites	1(1.9)
Inguinal node	1(1.9)
**Time between MRI and surgery, mean in days (ranges)**	7.64(2-15)
**Tumor type, No. (%)**	
Endometrioid	49(90.8)
Papillary serous	3(5.6)
Clear cell	1(1.9)
Adenosquamous	1(1.9)
**Tumor grade, No. (%)**	
G1	29(53.7)
G2	18(33.35)
G3	7(13)

Abbreviations: BMI; Body Mass Index, SD; Standard Deviation

**Table 4:** Correlation of MR Imaging with Histopathologic Results in 54 Patients

	Myometrial Invasion in Pathology			Total
	none	< 50%	≥ 50%	
**No myometrial invasion in MRI**	9	7	0	16
**< 50% myometrial invasion in MRI**	0	19	3	22
**≥ 50% myometrial invasion in MRI**	0	2	14	16
**Total**	9	28	17	54

Kappa test = 0.656

**Figure 2:**
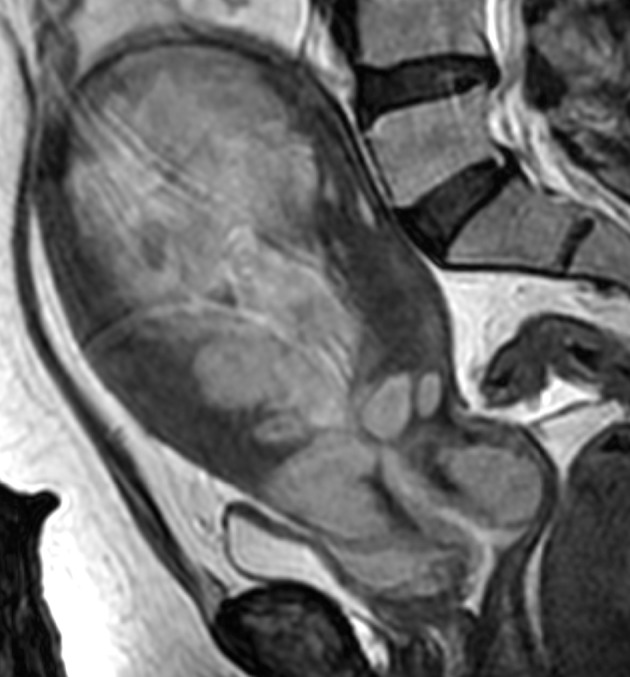
A 46-year-old woman with biopsy-proven endometrial carcinoma. Sagittal T2-weighted MRI shows a large mass within the endometrial cavity invading the deep myometrium and cervical stroma. Histopathology confirmed stage ІІ endometrial carcinoma.

According to the correlation of cervical invasion in MRI and the final pathology report, 29 out of 46 patients (63%) without cervical involvement in MRI did not have any cervical invasion in the surgicopthological report, 13 patients (28.3%) had only mucosal and four patients (8.7%) had stromal involvement. One out of two patients (50%) with mucosal involvement in MRI had mucosal and another one had stromal involvement (Figure 2). In case of cervical stromal involvement in the MRI report, all of them had stromal invasion in the pathology report (100% correlation between MRI and pathology). For cervical stromal involvement, the sensitivity, specificity, diagnostic accuracy, positive and negative predictive values and positive and negative likelihood ratios of MRI were 54.54%, 100%, 90.74%, 100%, 89.58%, 2.85 and 0.95, respectively (calculated with 95% confidence intervals). In the case of mucosal involvement, the positive predictive value was only 50% and the accuracy decreased to 74.07%. For agreement between MRI report and pathology of cervix, the Kappa was 0.347. According to old and new FIGO staging classifications, among the patients with stage Ia in MRI based on the old FIGO staging system, eight patients (57.1%) had the same pathological stage, two patients (14.3%) had Ib, one (7.1%) had IIa, one patient (7.1%) had IIb and two patients (14.3%) had IIIa. Six (85.7%) of the patients with Ic,had the same stage and one patient had stage IIa. Two (66.7 %) of the patients with stage IIb in MRI, had the same report and one (33.3%) had IIIa. The Kappa for agreement of old staging between MRI and pathology results was 0.628.

Based on the new FIGO staging, among patients who had stage Ia in MRI, 78.8% (26 patients) had the same staging, 6.1% (two patients) had Ib, 9.1% (three patients) had stage II and 6.1% (two patients) were in stage IIIa. All patients with stage Ib according to MRI had the same pathological stage. Two out of three patients with stage II according to revised FIGO staging in MRI had the same pathological staging and one had invasion to uterine serosa on pathology (IIIa). The Kappa for new staging between MRI and pathology was 0.72 (Table 5).

**Table 5:** Comparison of Diagnostic Performance Regarding Detection of Myometrial and Cervical Invasion in 54 Enrolled Patients

	N	TP	FP	FN	TN	Sen, %	Spe, %	Accuracy, %	PPV, %	NPV, %	PLR	NLR
**No Cervical Invasion**	54	29	17	0	8	100	32	68.5	63.0	100	1.4	0
**Cervical Mucus Invasion**	54	1	1	13	39	71.4	97.5	74.0	50	75	2.8	0.95
**Cervical Stromal Invasion**	54	6	0	5	43	54.5	100	90.7	100	89.5	**_**	0.45
**No Myometrial Invasion**	54	9	7	0	38	100	84.4	87.0	56.2	100	6.4	0
**< 50% Myometrial Invasion**	54	19	3	9	23	67.8	88.4	77.7	86.3	71.8	5.8	0.36
**≥ 50% Myometrial Invasion**	54	14	2	3	35	82.3	94.5	90.7	87.5	92.1	15.2	0.18

Abbreviations: FN; false negative, FP; false positive, NLR; negative likelihood ratio NPV; negative predictive value, PLR; positive likelihood ratio, PPV; positive predictive value, Sen; sensitivity, Spe; specificity, TN; true negative, TP; true positive

## 5. Discussion

Although modalities such as CT scan and MRI are not a part of FIGO staging for endometrial carcinoma, MRI can provide valuable data to estimate staging and choose the best treatment planning (9-11). The treatment of choice for endometrial cancer is surgery. Based on staging, cervical stromal involvement and the depth of myometrial invasion, the type of surgery can differ from a simple hysterectomy without lymphadenectomy or only a lymph node sampling to radical surgery with systematic lymphadenectomy (12, 13). MRI does not have any ionizing rays while it provides a high soft tissue contrast that makes it an optimal imaging technique for the female pelvis (14).

Moreover, with MRI we can decide about conservative management for patients who want fertility preservation or have medical problems that are high risk for surgery. It can be used for radiotherapy mapping too. The accuracy and sensitivity of MRI in the preoperation staging of endometrial cancer have been shown in several studies (15-17). Hirano et al. found that the accuracy, sensitivity, specificity, PPV and NPV in postcontrast MRI were 91%, 91%, 89%, 94% and 84%, respectively (15). Similar to other studies, in this analysis, the endometrioid type was the most common histology (18, 19). In our study, the majority of patients were in the early stage according to the final surgicopathology report (74.1% for stage I and II). The reported sensitivity of MRI in the diagnosis of cervical invasion for endometrial carcinoma had a range of 19-100% (20-28). A very low sensitivity (19%) reported in some studies may be due to the undetectable endocervical glandular involvement (stage IIA in the old system). In our study, the sensitivity and specificity in stromal invasion was higher in contrast to mucosal invasion diagnosis, 54.54% and 100% vs. 71.4% and 97.5%, respectively. These findings indicate the higher correlation of the new FIGO staging with MRI in the diagnosis of cervical involvement and its use in choosing the best treatment options. If there is a stromal cervical involvement, a radical surgery is the choice of treatment. The accuracy of MRI for diagnosing cervical mucosal involvement was very lower than cervical stromal involvement, 74.07% in contrast to 90.74%. The overall accuracy and specificity for cervical invasion ranged from 46-98% and 87-100 %, respectively (20-28). It has been proved that the depth of myometrial invasion has a direct relation with lymph node involvement and prognosis. If involvement of the myometrium is equal to 50% and more, the risk of lymph node involvement increases to more than 6-7 fold (13). Avoidance of unnecessary lymphadenectomy is important in low risk patients based on the depth of myometrial invasion, grading and histological subtype before surgery. So, knowledge about myometrial invasion is important for the extension and type of surgery (29, 30). It is indicated that MRI has the highest accuracy among the other imaging techniques in predicting the myometrial invasion (21-26, 28, 31, same as other studies 32). In a study performed by Riek et al. (31), there was a low sensitivity, accuracy and specificity, (47%, 36% and 50%, respectively) due to using an unenhanced MRI protocol. A meta-analysis revealed enhanced MRI is a better protocol for assessing myometrial invasion (9).We found an accuracy of 90.74% in our study for deep myometrial invasion. On the other hand, with a negative predictive value of 92.1%, this study indicates that MRI can predict deep invasion. This is in agreement with the results of other studies which found the accuracy of MRI for determining the depth of myometrial invasion ranging from 83% to 91% (17, 21-26, 28, 31, 33). Regarding staging and comparing the old and new FIGO staging in MRI and its correlation with surgicopathological findings, our results indicate a higher accuracy for the new staging classification. With a Kappa agreement of 0.72 for new and 0.62 for old staging, our results are as the same as other studies (21-26, 28). There are few works regarding the comparison of new and old staging. One of them indicates that the accuracy of MRI in new staging is more than the old system, but their study did not address myometrial and cervical involvement in detail (7). Thus, the present study could give useful data about MRI and endometrial cancer staging which does not have the limitation of such studies that have only assessed early stages of the disease. Furthermore, this work has all stages of endometrial cancer which results in lower sensitivity in contrast to the other studies.

Our study has some limitations. For example, uterine curettage could change the appearance of endometrial-myometrial interface on MRI. In addition, the interobserver agreement between the radiologists was not assessed in this study. Moreover, in certain conditions, it is difficult to assess the depth of myometrial invasion by MRI properly, for example in case of existence of the polypoid feature of the tumor, existence of a large uterine fibroma or adenomyosis, and when there is an isointense junctional zone (JZ) compared to the myometrium. In conclusion, MRI is a valuable imaging modality for presurgical staging of endometrial cancer, especially based on revised FIGO staging.
